# Increased oxidative stress in diabetes regulates activation of a small molecular weight G-protein, H-Ras, in the retina

**Published:** 2007-04-19

**Authors:** Vibhuti Kowluru, Renu A. Kowluru

**Affiliations:** Department of Ophthalmology, Kresge Eye Institute, Wayne State University, Detroit, MI

## Abstract

**Purpose:**

Increased superoxide levels are implicated in the pathogenesis of diabetic retinopathy. We have shown that functional activation of a small molecular weight G-protein, H-Ras, is one of the signaling steps involved in glucose-induced apoptosis of retinal capillary cells. The goal of this study was to elucidate the mechanism(s) by which oxidative stress could result in the activation of H-Ras in diabetes.

**Methods:**

Experiments were performed in isolated retinal endothelial cells that were treated with H_2_O_2_, or the cells in which glucose-induced superoxide accumulation was inhibited either by superoxide dismutase mimetic (MnTBAP) or by overexpressing mitochondrial superoxide dismutase (MnSOD). The in vitro experiments were complemented with in vivo experiments using the retina from mice overexpressing MnSOD.

**Results:**

H_2_O_2_ activated H-Ras and its downstream signaling pathway, including Raf-1 and phosphorylation of p38 (p-p38) MAP kinase. Inhibition of superoxide significantly attenuated glucose-induced activation of H-Ras, Raf-1 and p-p38 MAP kinase. Overexpression of MnSOD in mice prevented diabetes-induced activation of both H-Ras and p-p38 MAP kinase.

**Conclusions:**

Our results clearly indicate that the activation of H-Ras and its downstream signaling pathway in the retina and its vasculature could be under the control of superoxide, and H-Ras activation in diabetes can be prevented by inhibiting superoxide accumulation.

## Introduction

Diabetic retinopathy, a slow progressing complication of diabetes, is considered a multifactorial disease. Although many hyperglycemia-induced metabolic abnormalities are implicated in its pathogenesis, the exact mechanism of the development of retinopathy remains elusive. Our previous studies have suggested a role for the small molecular weight G-protein, H-Ras, in the development of retinopathy in diabetes. We have shown that functional activation of H-Ras is one of the signaling steps involved in glucose-induced capillary cell apoptosis. Inhibitors of H-Ras function reduce glucose-induced increased apoptosis of retinal capillary cells, and the therapy that inhibits apoptosis of retinal vascular cells and retinopathy in diabetes, inhibits diabetes-induced increase in Ras expression and mRNA levels in the retina [1-3].

Small molecular weight G-proteins act as one of the key regulators of the signaling cascade triggered by oxidative stress [4], and Ras is considered to be a common signaling target of reactive oxygen species (ROS) and cellular redox stress [5]. In addition, Ras-expressing cells produce high levels of superoxide [6], and superoxide production determines the sensitivity to intercellular induction of apoptosis [7].

Oxidative stress is elevated in the retina and its capillary cells and remains elevated when the histopathology can be seen in the vasculature [8,9]. Oxidative stress is closely linked to apoptosis in a variety of cell types, and could escalate apoptosis via increased membrane lipid peroxidation, injury to other macromolecules, or alterations in signal transduction [10,11]. Increased superoxide is considered to act as a causal link between elevated glucose and the major biochemical pathways postulated to be involved in the development of vascular complications in diabetes [12,13]. We have recently shown that inhibition of superoxide accumulation or overexpression of mitochondrial superoxide dismutase (MnSOD) inhibits diabetes-induced oxidative damage and apoptosis in the retina and its capillary cells [14-16]. The purpose of this study was to examine the putative mechanism(s) by which oxidative stress could result in the activation of H-Ras in retinal capillary cells. Using isolated retinal endothelial cells, we compared the effect of high glucose as well as H_2_O_2_ on the activation of H-Ras, and determined the effect of overexpression of MnSOD on glucose-induced activation of H-Ras and apoptosis. In vivo experiments were performed using the retina from the mice overexpressing MnSOD (MnSOD-Tg) to investigate the role of mitochondrial superoxide in the activation of retinal H-Ras in diabetes. The results presented demonstrate that superoxide regulate the activation of retinal H-Ras in diabetes.

## Methods

### Retinal endothelial cells

Retinal endothelial cells, prepared from cow eyes (obtained fresh from a local slaughterhouse), were cultured in Dulbecco's modified eagle medium (DMEM) containing 15% fetal calf serum (heat inactivated), 5% Nu-serum, heparin (50 μg/ml), endothelial growth supplement (25 μg/ml) and antibiotic/antimycotic in 95% O_2_ and 5% CO_2_ [1,2,14]. Confluent cells from third to sixth passage were incubated under normoglycemic (5 mM glucose) or hyperglycemic (20 mM glucose) conditions for 96 h in the presence or absence of 200 μM MnTBAP (a cell-permeable SOD mimetic, obtained from Biomol, Plymouth Meeting, PA), as described previously [14]. This duration was selected because our previous experiments have shown that mitochondrial dysfunction and apoptosis can be seen in retinal endothelial cells incubated in 20 mM glucose for over three days, and increasing the duration to 10 days does not increase the apoptosis [9,14,17]. Control incubations containing 20 mM mannitol were always run simultaneously to rule out the effect of increased osmolarity. Each experiment was repeated with at least three separate cell preparations.

### Incubation of endothelial cells with hydrogen peroxide

Hydrogen peroxide levels are elevated in the retina in diabetes [18]. In order to investigate the effect of increased hydrogen peroxide on H-Ras, its signaling pathway and apoptosis, we incubated endothelial cells from fourth to fifth passage with 250 μM H_2_O_2_ for 1 h. The cells were quickly washed with DMEM and incubated in 5 mM glucose and 20 mM glucose media for 96 h. At the end of the incubation period, the cells were washed with PBS, scraped, and used to determine the activation of H-Ras and phosphorylation of p38 MAP kinase, and cell apoptosis.

### Transfection of endothelial cells with MnSOD

Retinal endothelial cells were transfected with MnSOD expression plasmid DNA using a procedure described previously. In brief, 60-80% confluent endothelial cells from fourth passage were incubated with a transfection-complex comprising of MnSOD expression plasmid DNA and superfect transfection reagent (Qiagen,Valencia, CA) for 8 h. The transfected cells were then incubated in a fresh medium containing 2.5% fetal calf serum (heat inactivated), 10% Nu-serum, heparin (50 μg/ml), endothelial growth supplement (2.5 μg/ml) and antibiotic/antimycotic supplemented with 5 mM or 20 mM glucose for 96 h. As with our observations in previous studies, we found the efficiency of the transfection to be about 30% [16].

### Rodents

Hemizygous MnSOD-Tg mice, generated using human β-actin-MnSOD expression construct were bred with wild-type B6C3 F1 mice to generate experimental animals. The litters were genotyped by Southern blot analysis. A group of wild-type mice (WT) and MnSOD-Tg mice (body weight 18-22 g; male) were made diabetic by intraperitoneal injection of streptozotocin (60 mg/kg/body weight) for five consecutive days. We have shown that MnSOD Tg mice can be made diabetic successfully by giving similar doses of streptozotocin as their wild-type counterparts [16]. Body weight was determined once per week and blood glucose once every 10 days. Insulin (0.1-0.2 IU) was injected 2-5 times a week to prevent weight loss and ketonuria. Both diabetic and non-diabetic mice had free access to their food (standard laboratory Purina chow) and water. Their glycated hemoglobin (GHb) was measured at about eight weeks of diabetes, employing the affinity columns used by us previously [15]. Mice were sacrificed 3-4 months after induction of diabetes, and their retinas were isolated under a dissecting microscope. Treatment of the animals conformed to the Association for Research in Vision and Ophthalmology Resolution on the Use of Animals in Research.

### Activation of Ras by Raf-1 binding

Retinal endothelial cells incubated in either low (5 mM) or high (20 mM) glucose for 96 h were used to quantify the relative abundance of GTP-bound active H-Ras using a Raf-1 binding assay kit (Cytoskeleton, Denver, CO). This assay takes advantage of the high affinity of Ras-GTP for the Ras binding domain (RBD) of Raf-1. The cell extract was added to Raf-1RBD, and the Raf-RBD/GTP-Ras complex was pulled down by glutathione affinity beads. The beads were re-suspended in Laemmli reducing sample buffer and boiled for 5 min. The amount of activated Ras was determined by Western blot using Ras-Pan-specific antibody that was supplied in the Raf-1 binding assay kit.

### Protein expression of H-Ras and Raf-1

Protein (30-50 μg) was separated on a 12% denaturing polyacrylamide gel and transferred to nitrocellulose membranes. The membranes were blocked, followed by the incubation with the antibody against H-Ras or Raf-1 (Santa Cruz Biotechnology, Santa Cruz, CA). After incubation with horseradish peroxidase-conjugated secondary antibody, the membranes were developed using ECL-Plus Western blotting detection kit (Amersham Biosciences, Piscataway, NJ). Kaleidoscope pre-stained molecular weight markers (Bio-Rad Laboratories) were run simultaneously on each gel. To ensure equal loading among the lanes, we also determined the expression of the housekeeping protein, β-actin. The membranes were blotted for H-Ras or Raf-1, then incubated with stripping buffer (62.5 mM Tris-HCl pH 6.8, 100 mM mercaptoethanol, 2% sodium dodecyl sulfate) at 50 °C for 30 min, washed, and incubated with anti-β-actin (monoclonal antibody, Sigma Chemicals, St Louis, MO). The membranes were again washed. Next, they were incubated with horseradish peroxidase-conjugated secondary antibody, and developed using ECL-Plus Western blotting detection kit (Amersham Biosciences).Each band was quantified using Un-Scan-It Gel digitizing software (Silk Scientific Inc, Orem, UT), and the values in the histograms were presented as mean band density of the protein of interest (e.g., H-Ras, Raf-1) divided by the intensity of β-actin in the same sample.

### Activation of p38 MAP kinase

The activation of MAP kinase was determined by performing the Western blot analysis of the phosphorylation of p38 MAP kinase (a key signaling molecule for H-Ras-induced signaling pathway) using phospho-p38 (p-p38) antibodies from Cell Signaling Technology (Beverly, MA).

### Apoptosis

Endothelial cell apoptosis was determined using Cell Death Detection ELISA^PLUS^ kit from Roche Diagnostics (Indianapolis, IN). This method determines the relative amounts of mono- and oligonucleosomes generated from the apoptotic cells by using monoclonal antibodies directed against DNA and histones, respectively [1,15]. The cytoplasmic fraction of the cells was incubated with a mixture of peroxidase-conjugated anti-DNA and biotin-labeled anti-histone in a streptovidin-coated plate. The plate was washed thoroughly, incubated with 2,2'-Azino-di-[3-ethylbenzthiazoline sulfonate] diammonium salt (ABTS, Roche Diagnostics), and the absorbance was measured at 405 nm. The nuclear fraction obtained after separation of the cytoplasmic fraction was used to measure DNA content.

### Statistical analysis

Each experiment was repeated three or more times, and measurements were performed in duplicates. Values are reported as mean SD. The experimental groups were compared using the nonparametric Kruskal-Wallis test followed by the Mann-Whitney test.

## Results

### Effect of hydrogen peroxide on H-Ras, Raf-1, and MAP kinase activation and accelerated apoptosis

**Figure 1:**
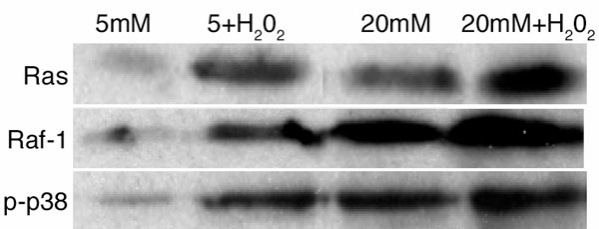
Effect of hydrogen peroxide on the activation of H-Ras and its signaling pathway. Bovine retinal endothelial cells were incubated with 250 μM H_2_O_2_ for 1 h. At the end of the incubation, the cells were rinsed with DMEM, and incubated in 5 mM glucose and 20 mM glucose media for 96 h. The activation of H-Ras was determined by Raf-1 binding assay, Raf-1 by measuring the expression of Raf-1 by Western blot, and that of MAP kinase by quantifying the expression of phospho-p38 of MAP kinase. Each sample was analyzed in duplicate, and the Western blots presented are representative of at least 3-4 experiments.

To determine if increased oxidative stress by itself could activate H-Ras and accelerate retinal cell apoptosis, we quantified activation of H-Ras (as measured by Raf-1 binding assay) and apoptosis in endothelial cells incubated with H_2_O_2_ in 5 mM glucose, and, for comparison, in 20 mM glucose medium. As shown in Figure 1, incubation of endothelial cells with H_2_O_2_ in 5 mM glucose medium activated H-Ras; the activation was further increased to 30-40% when the cells were incubated with H_2_O_2_ in 20 mM glucose medium. In the same cells H_2_O_2_ significantly activated Raf-1 and increased phosphorylation of p38 MAP kinase (Figure 1). The results obtained from the cells incubated with 20 mM mannitol for 96 h were not different from those obtained in 5 mM glucose medium (data not shown), suggesting that the effects of glucose were not due to the change in osmolarity.

**Figure 2:**
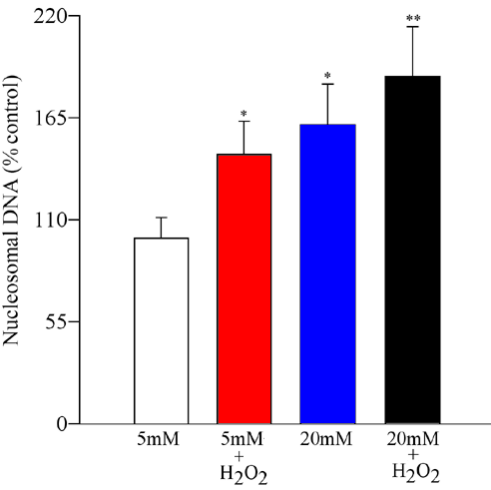
Effect of hydrogen peroxide on apoptosis of retinal endothelial cells. Apoptosis was measured in the endothelial cells pre-treated with H_2_O_2_, and incubated in 5 mM or 20 mM glucose for 96 h by performing ELISA for cytoplasmic histone-associated-DNA-fragments using an ELISA kit. The values are adjusted to the total DNA in each sample, and the numbers obtained from 5 mM glucose are considered 100%. Asterisk (*) refers to p<0.05 for 5 mM glucose, and double asterisk (**) indicates p<0.05 for 20 mM glucose.

Apoptosis of the retinal endothelial cells was increased by 45% when the cells were incubated with H_2_O_2_ in 5 mM glucose medium; however, when the cells were exposed to H_2_O_2_ in the presence of 20 mM glucose medium, cell death was increased to about 90% compared to the cells incubated in 5 mM glucose medium (Figure 2).

### Superoxide scavenging and glucose-induced Ras activation and its signaling steps in retinal endothelial cells

**Figure 3:**
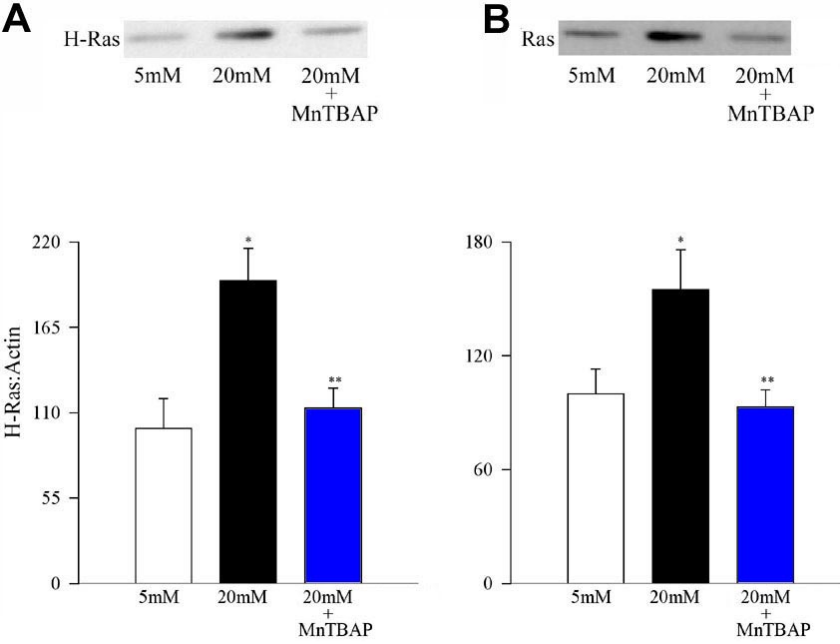
Effect of MnTBAP on glucose-induced activation of H-Ras. Endothelial cells were incubated in 5 mM glucose or 20 mM glucose medium for 96 h in the presence or absence of 200 μM MnTBAP for 96 h. Activation of H-Ras was estimated by (**A**) Western blot technique and Raf-1 (**B**) binding assay. Each experiment was repeated with at least three separate cell preparations. The histogram represents the ratio of the densities of H-Ras and β-actin in the same lane as quantified using Un-Scan-It gel software. The values obtained from the cells incubated in 5 mM glucose conditions are considered 100%. Asterisk (*) marks p<0.05 for 5 mM glucose, and double asterisk (**) denotes p<0.05 for 20 mM glucose.

As we previously reported [1], incubation of endothelial cells in 20 mM glucose resulted in the activation of H-Ras by over 50%, as determined by increased protein expression (Figure 3A) and also by Raf-1 binding (Figure 3B). However, when SOD mimetic, MnTBAP, was supplemented in the incubation medium, glucose-induced activation of H-Ras was significantly inhibited (p<0.05 compared to 20 mM glucose), and the values obtained from MnTBAP cells were not different from those obtained from the cells incubated in 5 mM glucose (p>0.05 compared to 5 mM glucose).

**Figure 4:**
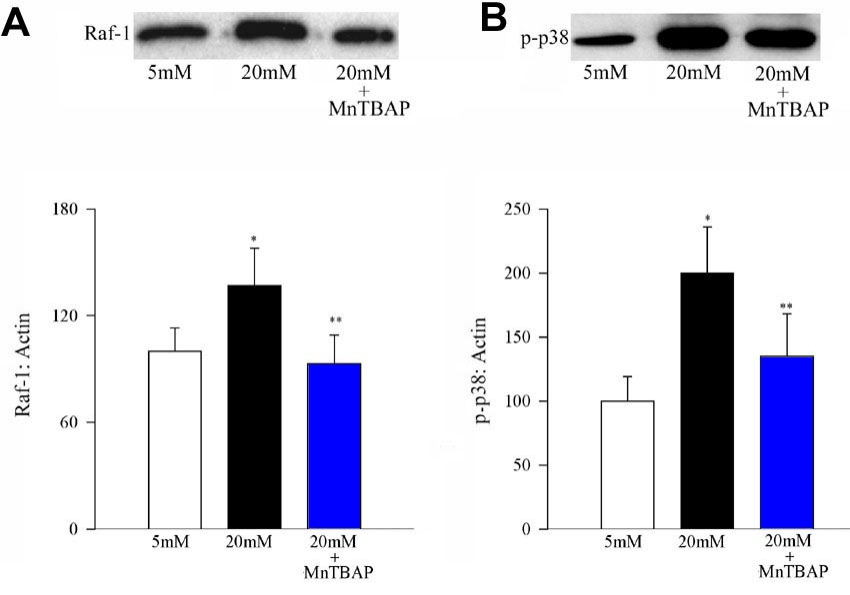
Effect of MnTBAP on glucose-induced activation of Raf-1 and phosphorylation of p38 MAP kinase. Activation of Raf-1 and phosphorylation of p38 MAP kinase were determined by Western blot using β-actin as a loading standard. Each sample was run in duplicate, and the experiment was repeated with three or more cell preparations. The histogram represents the density of Raf-1 (**A**), or p-p38 (**B**) band that has been adjusted to the density of β-actin band in the same lane. The ratio obtained from 5 mM glucose is considered as 100%. Asterisk (*) represent p<0.05 compared to untransfected cells in 5 mM glucose or MnSOD transfected cells incubated in 20 mM glucose, and double asterisk (**) marks p<0.05 compared to untransfected cells incubated in 20 mM glucose medium.

To investigate the effect of superoxide inhibition on the signaling steps that are under the control of H-Ras, we determined the activation of Raf-1 and phosphorylation of p38 MAP kinase in the same endothelial cells used in the work described in the previous paragraph. Figure 4A confirms the effect of high glucose on Raf-1 activation and Figure 4B shows glucose-induced phosphorylation of p38 MAP kinase in the retinal endothelial cells. As with H-Ras activation, MnTBAP also inhibited glucose-induced activation of both Raf-1 and phosphorylation of p38 MAP kinase (Figure 4A,B).

**Figure 5:**
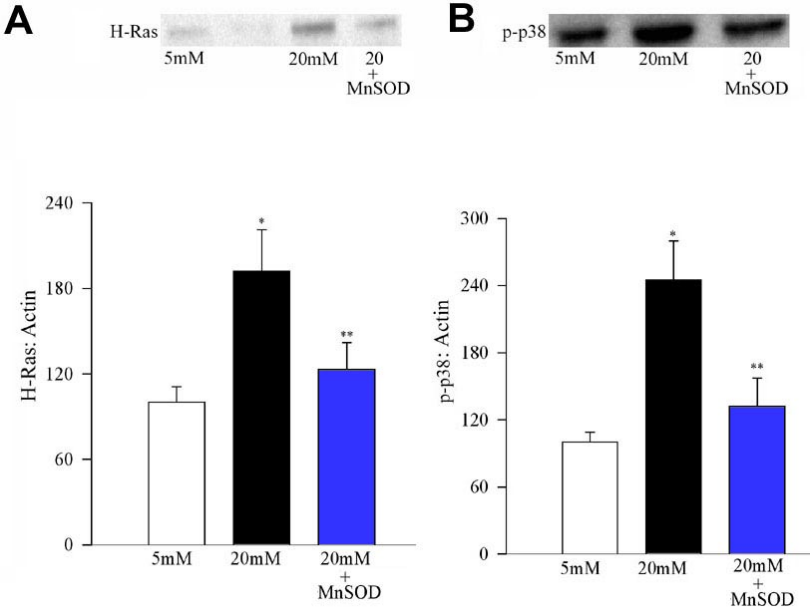
Effect of MnSOD overexpression on glucose-induced activation of H-Ras and MAP kinase. Bovine endothelial cells were transfected with MnSOD expression plasmid DNA using Superfect transfection reagent. After transfection, the cells were incubated in 5 mM or 20 mM glucose for 96 h. Activation of (**A**) H-Ras and (**B**) MAP kinase was determined by Western blot technique, and the band density was corrected for β-actin band density. Values obtained from the untransfected cells that were incubated in 5 mM glucose were considered to be 100%, and are mean±SD of three different transfection experiments. Please note that the lane in **A** between 5 mM glucose and 20 mM glucose is one of the lanes in which the molecular weight markers were loaded. 20 mM±SOD cells transfected with MnSOD that are incubated in 20 mM glucose medium. Asterisk (*) marks p<0.05 for 5 mM glucose, and double asterisk (**) indicates p<0.05 compared to 20 mM glucose.

To confirm the effect of superoxide on H-Ras activation and its downstream pathway, we used MnSOD overexpressing cells. Transient overexpression of MnSOD in the retinal endothelial cells inhibited glucose-induced activation of H-Ras (Figure 5A) and phosphorylation of p38 MAP kinase (Figure 5B) by over 60%. Activation of H-Ras and phosphorylation of p38 MAP kinase was similar in the normal (un-transfected) cells incubated in 5 mM glucose medium and MnSOD transfected cells incubated in 20 mM medium. In the transfected cells, overexpression of MnSOD prevented glucose-induced increase in mitochondrial superoxide levels (unpublished data). Thus our data presented here suggest that the regulation of H-Ras in endothelial cells could be under the control of mitochondrial superoxide.

### Overexpression of MnSOD in mice and diabetes-induced activation of H-Ras and MAP kinase in the retina

**Figure 6:**
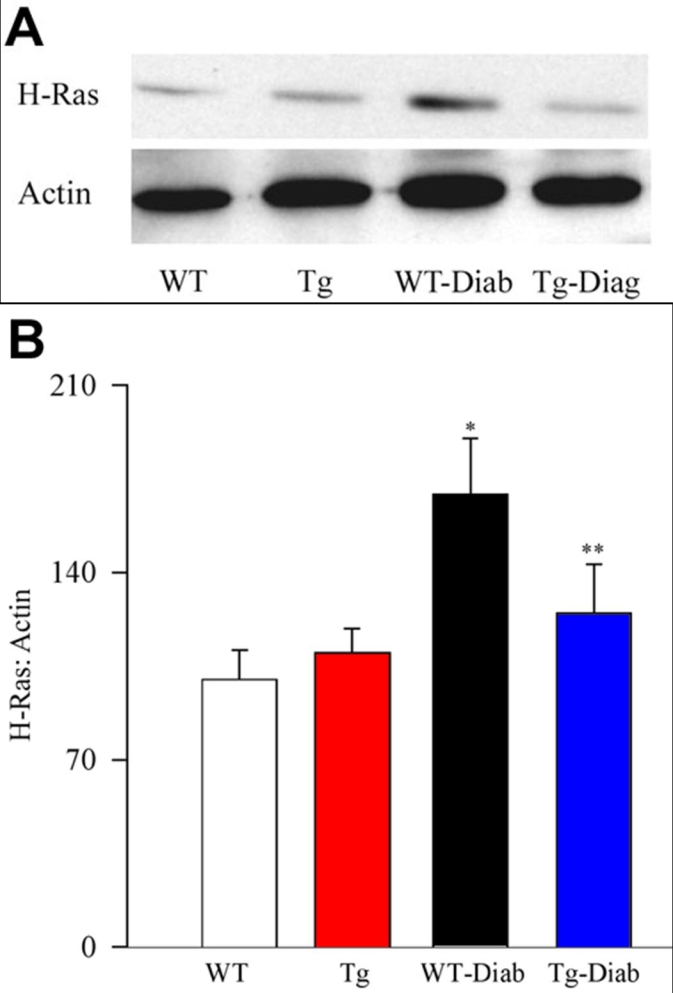
Effect of MnSOD overexpression on diabetes-induced activation of H-Ras in the retina of MnSOD-Tg mice. Activation of H-Ras was estimated in the retina of MnSOD-Tg and wild-type (WT) mice at 3-4 months of diabetes. Each retina sample was analyzed in duplicate, and (**A**) the Western blots represent five or more mice in each of the four groups. **B**: The histogram represents the ratio of the densities of H-Ras and β-actin in the same sample (quantified using Un-Scan-It gel software), and the ratio obtained from WT mice is considered to be 100%. Please note that the gel shows that the density of β-actin loading band is about 15-20% higher in diabetes, however, the ratio of the densities of the bands for H-Ras and β-actin in the same sample is about two-fold higher compared to other groups, and the mean H-Ras expression (adjusted to β-actin) obtained from five mice in each group (as depicted in the histogram) is over 1.7 fold higher in WT-diabetes compared to WT-normal mice. WT represents wild-type non diabetic; WT-Diab represents WT-diabetes; Tg represents MnSOD Tg-non diabetic; and Tg-Diab represents MnSOD Tg-diabetes. Asterisk (*) indicates p<0.05 compared to WT or Tg, and double asterisk (**) signifies p<0.05 compared to WT-Diab.

As shown in Figure 6, diabetes in mice results in activation of H-Ras in the retina. These results are in agreement with our previous results obtained from the retina of diabetic rats [1]. However, overexpression of MnSOD in mice protected the retina from diabetes-induced activation of H-Ras. The expression of H-Ras was only slightly higher in MnSOD-Tg diabetic mice (p>0.02) compared to non-diabetic WT or MnSOD-Tg mice; however the values were significantly different (p<0.05) compared to WT-diabetic mice.

**Figure 7:**
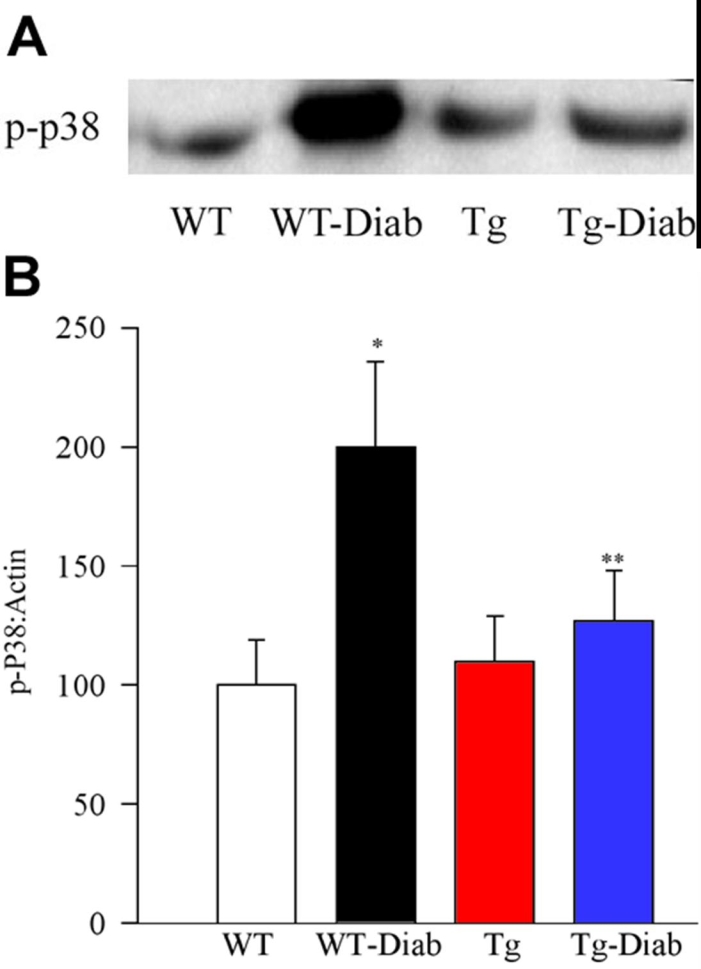
Effect of MnSOD overexpression on diabetes-induced activation of retinal MAP kinase. Activation of MAP kinase was estimated by measuring the expression of phospho-p38 in the same retina as used for H-Ras (**A**). The retina samples were analyzed in duplicate. The expression of β-actin in each row was used to correct the expression of phospho-p38 MAP kinase. **B**: The histogram represents the ratio of the densities of p-p38 and β-actin in the same sample (quantified using Un-Scan-It gel software), and the values obtained from WT mice are considered as 100%. Asterisk (*) signifies p<0.05 compared to wild-type (WT) or Tg, and double asterisk (**) indicates p<0.05 compared to WT-Diab.

Diabetes in WT mice increased phosphorylation of p38 MAP kinase by over 90% (Figure 7), but, in contrast, diabetes had no significant effect on the phosphorylation of p38 MAP kinase in the retina of MnSOD-Tg mice; the expression of p-p38 MAP kinase was similar in the retina obtained from MnSOD-Tg diabetic, non diabetic and WT-non diabetic mice (Figure 7).

Blood glucose was about four times higher in diabetic mice (mean values of 450 mg/dl in WT-diabetes and 410 mg/dl in MnSOD-Tg diabetes) compared to the non diabetic mice (mean values of 120 mg/dl in WT-non diabetic and 130 mg/dl in MnSOD-Tg non diabetic mice). Similarly, GHb values were also elevated by over 2.5 fold in diabetic mice, and the GHb values were comparable in WT and MnSOD-Tg mice: WT group had 5.3% and 13.8% GHb in non diabetic and diabetic mice, respectively, compared to 6.3 and 13.8 in the MnSOD-Tg non diabetic and diabetic mice. This clearly shows that the severity of hyperglycemia was similar in both WT-diabetic and MnSOD-Tg diabetic mice, which allowed us to investigate the effect of diabetes-induced oxidative stress, not the effect of hyperglycemia by itself, on the parameters of interest.

## Discussion

In diabetes H-Ras is activated, oxidative stress, including superoxide levels are elevated in the retina and its endothelial cells, and the therapies that inhibit the development of retinal histopathology in diabetes also inhibit Ras activation and superoxide accumulation [1,15,16]. In this study, we report that the activation of H-Ras and the downstream signaling pathway that can lead to capillary cell apoptosis in diabetes are under the control of superoxide accumulation in the retina. Induction of oxidative stress by exposure to H_2_O_2_ increased activation of H-Ras and its Raf-1-mediated downstream signaling pathway in retinal endothelial cells, and overexpression of MnSOD prevented diabetes-induced activation of H-Ras and its signaling steps. Incubation of endothelial cells with H2O2 has been shown to increase vascular endothelial growth factor (VEGF) levels and cell permeability, the parameters associated with diabetic retinopathy [18].

Diabetes-induced ROS act as a causal link between elevated glucose and the other metabolic abnormalities implicated in the development of complications [12,13]. We have shown that MnSOD plays a protective role in retinal capillary cell death, and ultimately, in the pathogenesis of retinopathy by protecting the retina from increased oxidative damage experienced in diabetic conditions [15,16]. We have observed similar superoxide levels in the retinal mitochondria obtained from diabetic and non diabetic MnSOD-Tg mice (unpublished data), suggesting protection by MnSOD. Furthermore, our recent studies have documented that the overexpression of the inactive mutant of H-Ras inhibits glucose-mediated increase in the apoptosis of retinal endothelial cells [17]. Here we provide evidence that inhibition of mitochondrial superoxide accumulation by either MnSOD mimetic or overexpression of MnSOD, results in the failure of high glucose to activate H-Ras in retinal endothelial cells. This suggests that the activation of H-Ras in diabetes could be under the control of mitochondrial superoxide. Our results are supported by others, showing that superoxide could modulate the downstream effects of Ras protein [19], and overexpression of SOD in Ras-transformed cells inhibits Ras-mediated transformation [20]. In contrast, overexpression of MnSOD has been shown to have no effect on Ras activation in hepatocytes [21]; the reason for such discrepancy is not clear.

Our exciting in vivo data demonstrate that in MnSOD-Tg mice the retina is protected from diabetes-induced activation of H-Ras. These results were obtained without ameliorating the severity of hyperglycemia in WT and MnSOD-Tg mice that were made diabetic with streptozotocin. This strongly supports our in vitro data obtained from the endothelial cells. Mitochondria dysfunction in the retina is associated with the pathogenesis of diabetic retinopathy; the release of cytochrome c into the cytosol and Bax into the mitochondria is increased in the retina in diabetes [9,14]. Bax immunostaining is seen in the retinal cells (vascular and ganglion) that are known to undergo accelerated apoptosis in diabetes [9,22]. Further, superoxide levels are elevated in the retina and in its endothelial cells in diabetes [8, and our unpublished data], and mRNA of superoxide dismutase is down regulated [16,23]. Overexpression of MnSOD in the retina protects it from diabetes-induced increase in oxidative stress and nitrative stress [15], and in the retinal endothelial cells prevents high glucose-induced increase in apoptosis [16]. ROS affect the interactions between Ras and several of its ligands [24], and ROS produced by advanced glycation-end products are shown to stimulate the Ras/Raf/MEK signaling cascade [25]. How Ras could be regulated by increased superoxide in diabetes is not clear. Other researchers have shown that the terminal cysteines of Ras are surface-exposed making them targets of oxidative or nitrosative processes [26,27], and oxidative stress and nitrative stress are elevated in the retina and its capillary cells in diabetes [8,9,28,29]. Moreover, Ras acts as a common signaling target of ROS and cellular redox stress [5], and Ras is considered to be one of the key regulators of the signaling cascade triggered by oxidative stress in human umbilical vein endothelial cells [4]. The targets for oxidizing agents are located both upstream and downstream of Ras, and ROS can affect the interactions between Ras and several of its ligands [24]. Thus, protection of diabetes-induced H-Ras activation in the retina by MnSOD strongly suggests that in diabetes H-Ras is regulated by mitochondrial superoxide.

Raf-1 is a key downstream effector protein of Ras function that in its inactive state is localized in the cytosol. We have shown that in diabetes, Raf-1 is increased in the retina. The therapies that inhibited the development of retinopathy in diabetic rats also inhibited Raf-1 activation [1]. Here we provide exciting in vitro and in vivo data, showing that inhibition of superoxide, either by superoxide mimetic or by overexpression of MnSOD inhibits diabetes-induced activation of Raf-1 in the retina. This clearly suggests that the Ras-mediated detrimental effects on retinal capillary cells in diabetes are via Ras-Raf-1 pathway.

Ras-dependent activation of Raf-1 can activate MAP kinase, and the MAP kinase family is considered to be an effector of the Ras-Raf pathway [8]. Phosphorylation of p38 MAP kinase is implicated in diabetes-induced increased apoptosis of retinal neuronal cells [30], and the mRNA expression of MAP kinase is increased in the retina as early as three days after induction of diabetes in rats [31]. Results presented in this manuscript clearly show that when H-Ras and Raf-1 activations are inhibited by inhibiting superoxide levels, p38 MAP kinase phosphorylation is also inhibited. This is confirmed by both our in vivo data from MnSOD-Tg mice and from the retinal endothelial cells overexpressing MnSOD. Activation of MAP kinase is dependent on the production of ROS, and phosphorylation of p38 as an important component of ROS-mediated signaling [32]. In diabetes, regulation of retinal vascular permeability by hepatocyte growth factor is considered to be mediated via MAP kinase pathway [33], and glucose has been shown to accelerate apoptosis of retinal endothelial cells via activation of MAP kinase [34]. Inhibition of diabetes-induced activation of H-Ras, Raf-1, and phosphorylation of p-p38 MAP kinase suggests that H-Ras activation in the retina could contribute to the development of diabetic retinopathy via activating Ras/Raf/MAP kinase signaling pathway.

In conclusion, this is the first report showing that the activation of H-Ras and its downstream signaling pathway in the retina and its vascular cells is under the control of superoxide. Our conclusion is supported by both in vivo (retina from MnSOD-Tg mice) and in vitro (retinal endothelial cells transfected with MnSOD) models. The results clearly demonstrate that H-Ras activation in diabetes can be prevented by overexpression of MnSOD, suggesting a complex cross-talk. The regulation of H-Ras-mediated signaling should help us identify novel pharmaco-therapeutic strategies for inhibition of retinopathy in diabetes.
